# Bridging the Postpartum Gap: A Randomized Controlled Trial to Improve Postpartum Visit Attendance Among Low-Income Women with Limited English Proficiency

**DOI:** 10.1089/whr.2020.0123

**Published:** 2021-09-09

**Authors:** Sarah Polk, Jill Edwardson, Shari Lawson, Doris Valenzuela, Elisabetta Hobbins, Laura Prichett, Wendy L. Bennett

**Affiliations:** ^1^ Department of Pediatrics, The Johns Hopkins University School of Medicine, Baltimore, Maryland, USA.; ^2^ Department of Obstetrics and Gynecology, The Johns Hopkins University School of Medicine, Baltimore, Maryland, USA.; ^3^ Oregon Health & Science University, Portland, Oregon, USA.; ^4^ Loyola University Chicago, Chicago, Illinois, USA.; ^5^ Division of General Internal Medicine, Department of Medicine, Johns Hopkins University School of Medicine, Baltimore, Maryland, USA.

**Keywords:** postpartum visit, maternal-child health, Latino, limited English proficiency, randomized controlled trial

## Abstract

***Background:*** Rates of postpartum visit attendance are low among all women, and particularly for low-income women. Experts in obstetrics, women's health, and health disparities are calling for novel, holistic approaches to postpartum care to better meet the needs of women and that respond to existing health care disparities.

***Materials and Methods:*** We conducted a single-site parallel-arm randomized controlled trial to determine the feasibility and effect of a co-located, co-timed 4–6 weeks postpartum obstetrics visit and well-newborn pediatric visit (*i.e*., “mommy-baby visit”) compared with an enhanced usual postpartum visit, that is, staff scheduled the postpartum visit for the patient before hospital discharge.

***Results:*** One hundred sixteen women, of whom 76.7% (*n* = 89) were Latina immigrants, were enrolled postdelivery and randomized to a mommy-baby visit (*n* = 58, 49.5%) or to enhanced usual care (*n* = 58, 50.4%). Almost all study participants attended their postpartum visit (*n* = 109, 94.0%). There was no significant difference in postpartum visit attendance rate by randomization assignment (91.4% of mommy-baby vs. 96.6% of enhanced usual care participants). Study participants, mommy-baby intervention and enhanced usual care arms combined, were significantly more likely to attend the postpartum visit than historical controls (94.0% vs. 69.7%, respectively, *p* < 0.001).

***Conclusions:*** In a randomized controlled trial, we showed postpartum visit attendance rates were high for participants in both the mommy-baby and enhanced usual care arms. Postpartum visit scheduling assistance was provided to all participants and may have increased postpartum visit attendance and thereby attenuated the effect of the intervention. It is encouraging that a low-cost, low-tech, low-touch intervention, that is, postpartum appointment scheduling before hospital discharge, could increase postpartum visit attendance.

## Introduction

Pregnancy provides an opportunity to identify and engage women at risk for future chronic disease because women are both more motivated than usual to make behavior changes to protect the health of their babies, and they are receiving ongoing, frequent prenatal care.^1^ The transition from prenatal care to ongoing preventive care could occur following delivery at the postpartum visit when clinicians might review the antepartum and intrapartum course, educate women about which chronic disease health risks arose during pregnancy, emphasize the importance of preventive care, and link women to available sources of ongoing preventive care.^2^

The postpartum visit is most critical for women with pregnancy complications like gestational diabetes mellitus or preeclampsia, which are known risk factors for type 2 diabetes and cardiovascular diseases.^3–6^ In addition, the majority of women in the United States enter pregnancy overweight or obese and/or gain excessive amounts of weight in pregnancy, and therefore need ongoing follow-up care, screening, and behavioral counseling to achieve a healthy body mass index.^7–9^

Despite the importance of the postpartum visit, rates of postpartum visit attendance are low among all women, and particularly for low-income women. Of the 199,860 Californian women with a Medicaid-funded delivery in 2012, only 33.3% of black women, 53.0% of Latina women, and 43.4% of white women attended a postpartum visit.^10^ Women report multiple barriers to receiving postpartum care, including childcare responsibilities, postpartum depression, and low levels of perceived personal risk for future illness.^11–13^ Based on experience in other settings, women with limited English proficiency (LEP), defined as speaking English less than “very well,” may particularly struggle to access postpartum care.^14^ LEP mothers have called it, “a battle,” to access care for their children.^15^ For adult patients, rates of both hospital admission and unplanned return visits to the Emergency Department within 48 hours were higher for LEP Spanish speakers than English speakers without LEP.^16^ In a study of pediatric emergency departments, LEP parents reported poorer care coordination compared to English-proficient parents.^17^

We report on a randomized controlled trial to determine the feasibility and effect of a co-located, co-timed maternal postpartum and newborn preventive care visit (“mommy-baby visit”) compared to an enhanced usual postpartum visit, that is, staff scheduled the postpartum visit for the patient before hospital discharge. Recruitment occurred between October 2015 and December 2016. We hypothesized that coordinating and co-locating women's postpartum care with their newborns' pediatric care would increase the rate of postpartum visit attendance. Our main aim of determining the feasibility and effect of the combined visits on postpartum visit attendance was nested within a long-term goal of enhanced ancillary support services addressing women's postpartum health and wellbeing funded by health care systems and/or by payers as an investment in population health. Our secondary aim was to collect data to strengthen the case to enhance postpartum care. For example, if engaging women in postpartum care increased their children's receipt of on-time primary care and immunizations, the justification for a population health-inspired investment in postpartum care visits would be strengthened. As such, our main outcome was postpartum visit attendance. Secondary outcomes included infant on-time receipt of well-child care and vaccines. Exploratory outcomes related to unmet maternal needs that might be addressed with ancillary services embedded in postpartum care, that is, maternal transition from prenatal care to ongoing primary care and maternal contraception.

## Materials and Methods

This trial was approved by the Johns Hopkins University School of Medicine Institutional Review Board.

### Study design

The “Bridging the Postpartum Gap: Mommy and Me Transitional Care” study was a single-site parallel-arm randomized controlled trial to determine the feasibility and effect of a co-located, co-timed 4–6 weeks postpartum obstetrics visit and well-newborn pediatric visit (*i.e*., “mommy-baby visit”) compared with an enhanced usual postpartum visit.

We designed the mommy-baby visit in collaboration with obstetricians, pediatricians, clinical managers, and the major Medicaid plan at our institution. We had identified postpartum visit attendance as an issue of collective interest to facilitate both the intervention's implementation and its sustainability. Hospital, health system, and managed care leaders are financially liable for postpartum visit attendance rates and often pay penalties due to low rates, while advocates for patient- and family-centered care consider the postpartum visit an underutilized opportunity to promote the health and wellbeing of mothers, children, and families.^18^ As such, the mommy-baby visit was integrated into a real care setting. The study involved both research and quality improvement components.

### Study setting

The study was conducted at a large academic hospital in Baltimore, MD. Participants were patients of the hospital's on-site obstetrics outpatient practice who had chosen the hospital's on-site pediatric outpatient practice for their newborn's care. The obstetric practice provides free prenatal care to low-income, insurance-ineligible local residents. The pediatric practice's majority patient population is publicly insured Latino children from immigrant families. The general pediatrics clinic averages 12,000 visits annually.

Baltimore is a new-emerging destination for Latino immigrants, who are the city's fastest growing ethnic group. The Latino population has nearly tripled since 2000, while Baltimore's overall population has decreased more than 8% over that same time period.^19–21^ LEP Latino families living in new-emerging destination states such as Maryland may find it especially difficult to navigate health care because of lack of information related to underdeveloped social networks in their communities and health care systems, which are ill-equipped to serve those with LEP.^22^

### Participant eligibility

Eligibility criteria included the following: (1) live birth, (2) discharge plan is for baby to go home with mother, (3) age >18, (4) English or Spanish speaking, (5) receipt of prenatal care at the delivery hospital, and (6) selection of the hospital-based pediatric practice for their child's care. Mothers of children in the neonatal intensive care unit (NICU) were excluded due to the unpredictability of NICU discharge and a desire not to further burden postpartum women managing the stress of a newborn in the NICU with the invitation to participate in a study. Women were excluded if they requested that an Intrauterine Device (IUD) be placed at the postpartum visit, as the postpartum visit was then misaligned with the 4-week well-newborn visit and we did not want study participation to interfere with any woman's access to her desired method of contraception, given limitations in postpartum care. Our obstetrician partners requested the IUD-related exclusion and it was important to address their patient care priorities in implementing the pilot intervention.

### Study recruitment

**FIG. 1.**
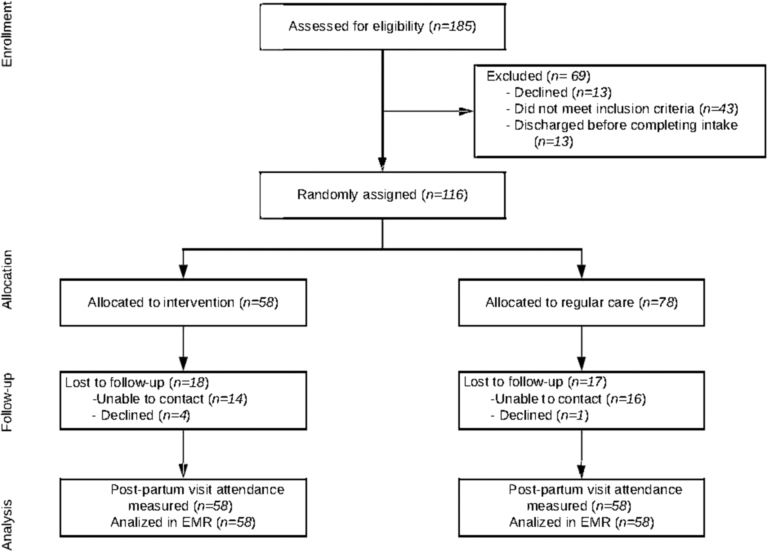
Study consort diagram.

The day before recruitment, study staff contacted postpartum unit nursing staff to ask which patients would be ready for discharge from the postpartum unit the next morning (Fig. 1). On the day of recruitment, participants were screened for eligibility using the electronic medical record. When unclear, research study staff asked postpartum unit nurses to confirm eligibility. Study staff then asked the nurse caring for the eligible patient to request the patient's permission to approach the patient about the study. When the patient agreed, the trained study staff member introduced the study and then, for interested patients, confirmed eligibility, consented, and randomized the participant. Recruitment occurred between October 2015 and December 2016.

### Active intervention

Following randomization, participants in the mommy-baby visit arm were scheduled for a co-located, co-timed postpartum/well-child visit at 4 weeks after delivery designed through a partnership between obstetrics and pediatrics. The pediatricians performed the infant's checkup either in the same patient room as the mother's obstetric visit or in the adjacent pediatric practice immediately before the postpartum visit. We created paired appointment slots in the obstetrics and pediatrics practices for study participants at the end of the morning session. Four weeks was chosen as the time of the joint visit because, while many newborns have several visits with the pediatrician between birth and 4 weeks, those visits are unpredictable and very time sensitive. All newborns are recommended to have a visit at 4 weeks, making that an ideal joint visit for logistical purposes. When possible, based on insurance eligibility and participants' wishes, study staff arranged a new or follow-up primary care visit within 3 months of the postpartum visit in the hospital-based General Internal Medicine practice.

### Control group intervention

Women randomized to the control group received usual postpartum care, separate maternal and child visits. Their usual care was “enhanced” in that study staff scheduled participants' postpartum visits before hospital discharge. We scheduled control group postpartum visits to remove scheduling as a confounder.

### Historical controls

We conducted a medical record abstraction to determine postpartum visit attendance in the 1 year before our study for women who both received prenatal care at the outpatient obstetrics practice and chose the co-located outpatient pediatric practice for the care of their newborn. A total of 453 mothers established care at the pediatrics practice during the 12 months before the start of the study. We used a random number generator to select the random sample of 175 charts (40%). Of those 175 newborns, 155 of their mothers had received prenatal care at the co-located obstetric practice. We then documented whether or not the mother attended a postpartum visit.

### Outcomes

We performed electronic medical record abstraction to determine the main outcome, postpartum visit attendance. Secondary outcomes were infant on-time receipt of well-child care and vaccines. Exploratory outcomes were proportion of women who attended a primary care visit in our hospital system in the 12 months after delivery and who initiated contraception postpartum.

### Data collection

Data collection included an in-person, staff-administered questionnaire after delivery (baseline) and by phone at 6 and 12 months postpartum. Standard questionnaires consisted of multiple choice and Likert-scale questions in English or Spanish concerning sociodemographics, health care utilization, and contraception. Regarding contraception, long-acting reversible contraception (LARC) included intrauterine devices and implantable contraception. Staff administered surveys to accommodate variation in participant literacy.

Electronic medical record abstraction was used to determine health insurance status and payer, medical history, pregnancy, labor and delivery complications, infant well-child care and vaccination, and maternal postpartum primary care.

### Statistical analysis

We estimated a baseline postpartum visit attendance rate of 70% based on prior data for members of the Medicaid Managed Care Organization insuring most of the pediatric clinic's patients. Assuming a type 1 error tolerance of 0.05, 62 participants per arm was estimated to provide 80% power to detect an absolute difference of 20% between the intervention and control group postpartum visit attendance rates (*i.e*., an increase from 70% to 90% in the intervention group).

We used descriptive statistics to compare the baseline characteristics of the two study arms. We used chi-square or analysis of variance tests (depending on whether a measure was binomial or continuous) to investigate differences in the proportions of women who attended postpartum visits, as well as to assess the by-arm differences in the other outcomes, that is, the proportion of women who initiated contraception, attendance at a primary care visit in the 12 months after delivery, and receipt of newborn vaccinations.

## Results

A total of 116 women were enrolled postdelivery and were randomized to mommy-baby visit (*n* = 58, 49.5%) or to enhanced usual care (*n* = 58, 50.4%) (Fig. 1).

There were no statistically significant differences in participants' baseline demographic characteristics by randomization assignment (Table 1). Mean age was 27.7 years. Approximately one-third of participants were nulliparous (31.0% mommy-baby and 29.3% usual care). Most participants were Latina (82.8% mommy-baby and 79.3% usual care). Of non-Latina participants, 10.4% were black (10.3% mommy-baby and 8.6% usual care) and 6.9% were white (6.9% mommy-baby and 6.9% usual care). All Latina participants received free prenatal and postpartum care through the combination of a charity care program for low-income, insurance-ineligible women and Emergency Medicaid. Most non-Latina participants (90%) were enrolled in Medicaid, although 10% had private insurance.

**Table 1.** Demographic and Health Care Utilization Characteristics of Maternal Participants in Pilot Trial of Co-Scheduled Postpartum and Newborn Well-Care Visits

	Mommy-baby group, *n* = 58, *n* (%)	Usual care group, *n* = 58, *n* (%)
Age, mean (SD)	27.7 (5.7)	27.7 (6.3)
Race/ethnicity		
Hispanic	48 (82.8)	46 (79.3)
Black, non-Hispanic	6 (10.3)	5 (8.6)
White, non-Hispanic	4 (6.9)	4 (6.9)
Other	0	3 (5.2)
Married or living with partner	50 (87.7)	52 (89.7)
Educational attainment		
≤6th Grade	18 (31.0)	14 (24.6)
7th to 12th Grade	18 (31.0)	20 (35.1)
High school or GED	15 (25.9)	17 (29.8)
Some college	1 (1.7)	4 (7.0)
College	6 (10.3)	2 (3.5)
Health insurance		
Medicaid	12 (21.8)	11 (19.6)
Hospital-based charity care	42 (76.4)	42 (75.0)
Private insurance	1 (1.8)	3 (5.4)
Parity (before delivery)		
Nulliparous	18 (31.0)	17 (29.3)
1–2 Births	32 (55.2)	36 (62.1)
3–4 Births	8 (13.8)	5 (8.6)
Pregnancy complications		
Gestational diabetes	4 (6.9)	6 (10.3)
Preeclampsia	1 (1.7)	0
Preterm birth	1 (1.7)	3 (5.2)
Health care utilization		
Received early prenatal care	26 (44.8)	23 (36.7)
Had checkup in 12 months before pregnancy	9 (15.5)	14 (24.1)
Additional demographic characteristics of Latina participants, *n* = 94, *n* (%)		
Country of birth		
Honduras	38 (40.4)	
El Salvador	27 (28.7)	
Mexico	15 (16.0)	
United States	5 (5.3)	
Other	9 (9.6)	
Time in the United States (if born outside United States)		
<1 Year	7 (7.5)	
Longer than 1 year	84 (89.4)	
Preferred language is Spanish	87 (92.6)	
English Proficiency (if not preferred language)		
Very well	5 (5.3)	
Not well	41 (43.6)	
Not at all	43 (45.7)	
Education		
≤6th Grade	32 (34.0)	
7th to 12th Grade	30 (31.9)	
High school or GED	27 (28.7)	
Some college	2 (2.1)	
College	2 (2.1)	
Do not know/blank	1 (1.0)	
Health insurance		
Medicaid	7 (7.5)	
Hospital-based charity care	86 (91.5)	
Private insurance	1 (1.0)	

SD, standard deviation.

Almost all Latina participants were foreign born (*n* = 89, 94.6%) and 89% had been in the United States for more than 1 year. As is typical in Baltimore City, they represented a variety of countries of origin: 40% Honduras, 29% El Salvador, 16% Mexico, and 15% other. Consistent with the fact that 93% reported Spanish as their preferred health care language, 89% reported LEP. Most (66%) had less than a high school education.

Almost all participants in both the mommy-baby and enhanced usual care arms attended their postpartum visit (*n* = 109, 94.0%). There was no significant difference in postpartum visit attendance rates by randomization assignment (91.4% of mommy-baby vs. 96.6% of enhanced usual care participants) (Table 2). Mommy-baby intervention and enhanced usual care arms combined were significantly more likely to attend the postpartum visit than historical controls (94.0% vs. 69.7%, respectively, *p* < 0.001).

**Table 2.** Postpartum Visit Attendance by Randomization Assignment and by Select Maternal Characteristics Participants in Pilot Randomized Trial of Co-Located and Co-Scheduled Postpartum and Newborn Visits

	Mommy-baby group, *n* = 58, *n* (%)	Usual care group, *n* = 58, *n* (%)
Overall	53 (91.4)	56 (96.6)
Race/ethnicity		
Hispanic	45 (84.9)	44 (78.6)
Non-Hispanic	8 (15.1)	12 (21.4)
Insurance type		
Charity care	39 (78.0)	41 (75.9)
Public	10 (20.0)	10 (18.5)
Private	1 (2.0)	3 (5.6)
Received early prenatal care	23 (43.4)	23 (41.1)
Any pregnancy complication	24 (45.2)	23 (41.1)

*p* > 0.05 for all fields.

Regarding secondary outcomes (Table 3), most infants had on-time receipt of vaccines regardless of randomization assignment (71.4% mommy-baby and 66.1% usual care). Regarding exploratory outcomes, the majority of participants received long-acting reversible contraception or tubal ligation before discharge from the hospital postpartum (65.5% mommy-baby and 60.3% usual care). Few women had a primary care visit in the 12 months after delivery (17.2% mommy-baby and 13.8% usual care).

**Table 3.** Secondary Outcomes of Maternal and Infant Participants in Pilot Randomized Trial of Co-Located and Co-Scheduled Postpartum and Newborn Visits

	Overall, *n* = 116	Mommy-baby group, *n* = 58	Usual care group, *n* = 58
Infant outcomes			
On-time well-child care, *n* (%)	54 (48.2)	29 (51.8)	25 (44.6)
On-time receipt of vaccines, *n* (%)	77 (68.7)	40 (71.4)	37 (66.1)
No. of acute care visits, mean (SD)	1.26 (1.56)	1.18 (1.57)	1.35 (1.57)
No. of ED visits, mean (SD)	1.30 (1.68)	1.46 (1.80)	1.12 (1.56)
Maternal outcomes, *n* (%)			
Contraception			
LARC^a^ started or surgery before discharge	73 (62.9)	38 (65.5)	35 (60.3)
Initiating/continuing contraception at postpartum visit	91 (78.4)	42 (72.4)	49 (84.5)
Using LARC at 6 months	69 (59.5)	33 (56.9)	36 (62.1)
Using LARC at 12 months	70 (60.3)	32 (55.2)	38 (65.5)
Health care utilization, *n* (%)			
Primary care visit within 12 months after delivery	18 (15.5)	10 (17.2)	8 (13.8)

*p* > 0.05 for all fields.

^a^ LARC includes IUD, injectable, implant, tubal ligation, and vasectomy.

ED, Emergency Department; IUD, Intrauterine Device; LARC, long-acting reversible contraception.

## Discussion

In this randomized controlled trial of a co-located, co-timed 4–6 weeks postpartum obstetrics visit and well-newborn pediatric visit (*i.e*., “mommy-baby visit”) compared with an enhanced usual postpartum visit, we showed postpartum visit attendance rates were high (>90%) for participants in both the mommy-baby and enhanced usual care arms. We also demonstrated that co-timed, co-located maternal-newborn postpartum visits are a feasible and acceptable health service intervention. Study participants in both arms were significantly more likely to attend their postpartum visit than historical controls. Study staff scheduled postpartum visits for all participants, both in intervention and control, which may have attenuated the effect of the intervention.

Regarding secondary outcomes, on-time infant well-child care was slightly lower (71.4% mommy-baby and 66.1% usual care) than in a more intensive randomized controlled trial conducted in the same practice (85% active intervention and 79% usual care).^23^ Two exploratory outcomes of interest were maternal postpartum contraception and maternal transition to ongoing preventive care in the year following delivery.

The postpartum visit is cited as a critical opportunity to promote healthy birth spacing. Our study was not designed to increase rates of LARC or other contraception and the participants randomized to a mommy-baby visit did not have higher rates of LARC or other contraception. The lack of impact on postpartum LARC use in this study may be due to the high percentage of participants who had received LARC before hospital discharge (*n* = 73, 62.4%) and that patients scheduled for IUD insertion postpartum were ineligible to participate.

In our study, although almost all participants attended their postpartum visit, few had a primary care visit within 12 months postpartum, while 8.7% (*n* = 10) had gestational diabetes, which has been shown to significantly increase the risk of future cardiovascular disease independent of future type 2 diabetes.^24^ The same women are at risk both of postpartum visit nonattendance and of future chronic disease, especially cardiovascular disease, that is, low-income, African American and Latina women.^25^ Citing an “urgent need to reduce severe maternal morbidity and mortality,” The American College of Obstetricians and Gynecologists has called for an overhaul of the postpartum visit to reduce future risks and to enhance care coordination and the primary care transition.^2^ The proposal includes replacing a single visit at 6 weeks postpartum with a series of visits culminating in a visit at 12 weeks to formally transition women into ongoing primary care. The perinatal period is an important time to engage women in care for the prevention of chronic disease as women are especially receptive to behavior change. The adoption of healthy behaviors would benefit the mothers and might spillover to their children who share their genetic, socioeconomic, and environmental risks for future disease.

Our study had some noteworthy strengths. Most (97%) women approached for study participation agreed to join the study. The study population consisted almost exclusively of low-income women who are less likely to receive postpartum care.^26^ Latino ethnicity and lack of insurance are associated with postpartum visit nonattendance.^27^ The study population included LEP Latinas who face obstacles to health care access and also have high future risk of chronic disease. Our findings may be of particular relevance in new destination cities for Latino immigrants.

Several limitations of this randomized controlled trial deserve mention. First, we did not achieve our recruitment goal of *n* = 62 in each arm due to time constraints, limiting the power to detect a between group difference. However, postpartum attendance rates were so much higher than anticipated in both groups that it would be unlikely to detect a significant difference. Second, we had relatively low 6- and 12-month follow-up rates, and the participants who responded at those times may not be representative of the entire study population. Finally, these findings are from a single site, which may limit their generalizability.

Several next steps are planned or underway. First, given that our findings suggest assistance with appointment scheduling may be quite valuable to this population, we will test the impact of enhanced discharge planning. Second, we have begun a trial of group well-child care for children of Latina immigrants as a means of providing more maternal- and family-centered care in a pediatric primary care setting. Third, we integrated a community health worker into our pediatric practice to address maternal questions and unmet needs in regard to family planning. We suggest that there is a need for synergy between women's and children's health care to capitalize on ongoing conversations between women and health care providers of topics relevant to child, parent, and family wellbeing, for example, family planning and maternal mental health. Specifically, we suggest our findings complement and extend the March of Dimes IMPLICIT (Interventions to Minimize Preterm and Low birth weight Infants using Continuous Improvement Techniques) Interconception Care Toolkit from family medicine to pediatrics, another important health care setting for children and families.^28^ Finally, additional research is warranted to determine how to make postpartum visits more relevant and valuable to women's overall health and well-being. Maryland, where this trial was conducted, just expanded Medicaid coverage for pregnancy from 8 weeks postpartum to 12 months postpartum (Senate Bill 923), which may offer a way to better and more holistically attend to women's postpartum health and health care needs.^29^ This Medicaid expansion excludes undocumented immigrants, however, thereby threatening to increase existing disparities.

Difficulty scheduling the postpartum visit has been reported as a barrier to visit attendance in other studies.^30^ It is encouraging that a low-cost, low-tech, low-touch intervention could increase postpartum visit attendance alone, as we showed in the control group. Scheduling postpartum visits before discharge may be especially valuable for LEP patients in new destinations for LEP immigrants, but may be helpful even for those without LEP. Newborns cannot be discharged without having a primary care visit scheduled. Maybe the same standard should apply to the postpartum visit for women? Additional modifiable barriers to postpartum care deserve attention, including childcare responsibilities, postpartum depression, and low levels of perceived personal risk for future illness.^11–13^ Womens' health advocates have proposed a variety of solutions to modifiable barriers, including enhancing health care teams with community health workers.^31^ Addressing barriers to health care access for low-income minority women is important to avoid perpetuating or exacerbating health and health care disparities.

## Abbreviations Used

LARC: long-acting reversible contraception
LEP: limited English proficiency
NICU: neonatal intensive care unit
SD: standard deviation
